# Barriers and facilitators for strengthening physiotherapy services in Nepal: perspectives from physiotherapists and health providers

**DOI:** 10.1186/s12913-024-11272-w

**Published:** 2024-08-01

**Authors:** Nishchal Ratna Shakya, Amanda Emén, Gillian Webb, Hellen Myezwa, Biraj Man Karmacharya, Ann-Katrin Stensdotter

**Affiliations:** 1 Department of Neuromedicine and Movement Science, Faculty of Medicine and Health Sciences, Norwegian University of Science and Technology (NTNU), Trondheim, 7491 Norway; 2 Department of Public Health and Nursing, Faculty of Medicine and Health Sciences, Norwegian University of Science and Technology, Trondheim, Norway; 3 Department of Physiotherapy, Kathmandu University School of Medical Sciences, Dhulikhel, Kavre, Nepal; 4 Faculty of Medicine, Dentistry and Health Sciences, The University of Melbourne, Parkville, Australia; 5 School of Therapeutic Sciences, University of the Witwatersrand, Johannesburg-Braamfontein, Gauteng, South Africa; 6 Department of Public Health, Kathmandu University School of Medical Sciences, Dhulikhel, Kavre, Nepal

**Keywords:** Health system, Disability, Non-communicable disease, Chronic diseases, Socio-ecological model

## Abstract

**Background:**

Physiotherapy provides non-invasive and non-pharmaceutical intervention for curative, rehabilitation and preventative purposes. Physiotherapy is also a central provider of health promotion. As the global burden of non-communicable diseases and chronic health conditions is rising, the importance of physiotherapy services increases. Unfortunately, physiotherapy services in low- and middle-income countries (LMICs) are generally unsatisfactory. In Nepal, the earthquake in 2015 and the COVID pandemic have clearly illuminated the importance of physiotherapy.

**Objective:**

This qualitative study aimed to identify barriers and facilitators at different system levels for strengthening physiotherapy services in Nepal.

**Methods:**

Forty semi-structured individual interviews were performed with different health providers. Transcribed interviews were assessed with thematic analysis. A five-level socioecological framework conceptualised multilevel determinants of barriers and facilitators.

**Results:**

The study revealed various factors that were potential barriers and facilitators across five different levels, namely individual (taking the lead, need for advocacy), interpersonal (lack of recognition and autonomy, networking for referrals and coordination), community (lack of knowledge and awareness, social and family support), organisational (accessibility, workplace and clinical practice, educational opportunities, role of organisations and rehabilitation centres), and public policy level (planning and implementation of policies and programs, medical hegemony, priorities). Government officials, local leaders, and clinicians, half of whom were physiotherapists, agreed on many of the same issues, where a lack of awareness of what physiotherapy is and knowledge about what physiotherapists do was central.

**Conclusions:**

The results provide information for the development of physiotherapy by pointing out key elements that need attention. Our broad and structured investigation strategy is applicable to others for a comprehensive analysis of barriers and facilitators for physiotherapy services.

**Supplementary Information:**

The online version contains supplementary material available at 10.1186/s12913-024-11272-w.

## Introduction

The health system of Nepal faces many challenges. One is the disproportionate lack of human resources relative to the increasing burden of chronic health conditions and growing elderly populations [1]. Among the 35 registered health professions under the Nepal Health Professional Council (NHPC) [2], physiotherapy plays an important role [3]. Physiotherapists work in most medical fields across the entire healthcare system according to a biopsychosocial concept adopting the International Classification of Function [4, 5]. The number of physiotherapists per 1000 is considerably higher in some Asian countries and Europe where Norway has the highest number [6], however low- and middle-income countries (LMICs) lag behind. In Nepal, the estimated number is 0.08 per 1000 [7]. To mitigate the deficit in manpower, Nepal has a small but growing educational capacity [8]. Despite the obvious deficit, many physiotherapists struggle to find employment [9]. There is thus an unresolved discrepancy between needs, understaffing, and unemployment [10].

The estimated prevalence of disability worldwide adds up to over one billion where a considerable number of individuals would benefit from rehabilitation [11, 12]. This indicates an increasing demand globally where LMICs such as Nepal, face an urgent need to strengthen rehabilitation and health-services. Physiotherapy has an important role endorsing health promotion and injury prevention [3].

Physiotherapists play a leading role important in the development of rehabilitation [13]. In Nepal, integration of physiotherapy services into the healthcare system is however poor or altogether absent [14]. With the promulgation of a new constitution in 2015, Nepal became a federal democratic republic with three levels of government: a federal level, seven provinces, and 753 local governments assuring health as a fundamental right of every citizen [15]. Nepal has ratified the United Nations sustainable development goals and Universal Health Coverage (UHC) [16] and has in the last decade achieved significant progress in various public health initiatives [17]. Nevertheless, 33.92% of the rural population [18] have difficulty in accessing health services [19]. Nepal is also one of the most disaster-prone countries in the world due to its topography and climatic circumstances [20]. In 2015, two consecutive earthquakes struck Nepal, each exceeding seven on the Richter scale [21] followed by hundreds of aftershocks [22], that left 8897 dead and 22,310 injured [23, 24]. Earthquake victims together with the aftermath of the Covid-19 pandemic [25], the increase of non-communicable diseases [26], and injury incidents [27] add up to a high number of people in need of rehabilitation [28]. The government has also committed to provide medical treatment and rehabilitation to those who suffered disabilities during the 1996–2006 conflict period [29]. These circumstances emphasize a considerable and urgent requirement for physiotherapy and rehabilitation services as evident also in other LMICs [30, 31].

Despite recent significant support from the government for improving human resources in the health sector, physiotherapy has not yet been recognised as a priority. Due to the importance of advancing the rehabilitation sector, health policy- and health systems research is considered a high priority [32]. In Nepal, such studies are lacking. The objective of the present study was therefore to identify factors serving as possible facilitators and barriers to the development of physiotherapy as an integral part in the health service system in Nepal.

### Theoretical framework

To gain information in a new and uncharted area, we considered interviews of health providers suitable, using thematic analysis for identifying facilitators and barriers. For a comprehensive view across system levels from person to policy, a socioecological model (SEM) was applied as a framework [33]. This model encompasses a broad, multidisciplinary perspective spanning five different system levels demonstrating the interactive effect and influence of factors at and between each level [34–36]. The *individual level* concerns personal determinant*s* about knowledge, attitude, perception, and skills. The *interpersonal level* is about the influence of personal relationships. The *community level* refers to the influence of environmental determinants. The *organisational level* consists of institutional rules and regulations affecting operations. The *public policy level* refers to local, state, and national legislatures, governing bodies, and actions (Fig. 2). As different factors and determinants influencing the development of physiotherapy can exist at all system levels, a comprehensive view implies that all SEM levels must be simultaneously addressed for impact [37].

## Methods

### Study design

A cross-sectional interview study was conducted using a stratified, purposive sampling approach to attain a variation of perspectives [31, 38, 39] at different systems level. Semi-structured in-depth individual interviews were used to optimise interview time for systematic and comprehensive exploration while keeping the interview focused on the desired line of action [40]. The first round, including only physiotherapists, was insufficient to populate all SEM levels. A second round of interviews was thus undertaken with other health professionals and authorities in the health sector. Emerging issues occurring in the interviews were identified with additional interviewees recommended by participants [41]. Consolidated criteria for reporting qualitative research (COREQ) was followed [42].

### Participants

**Fig. 1:**
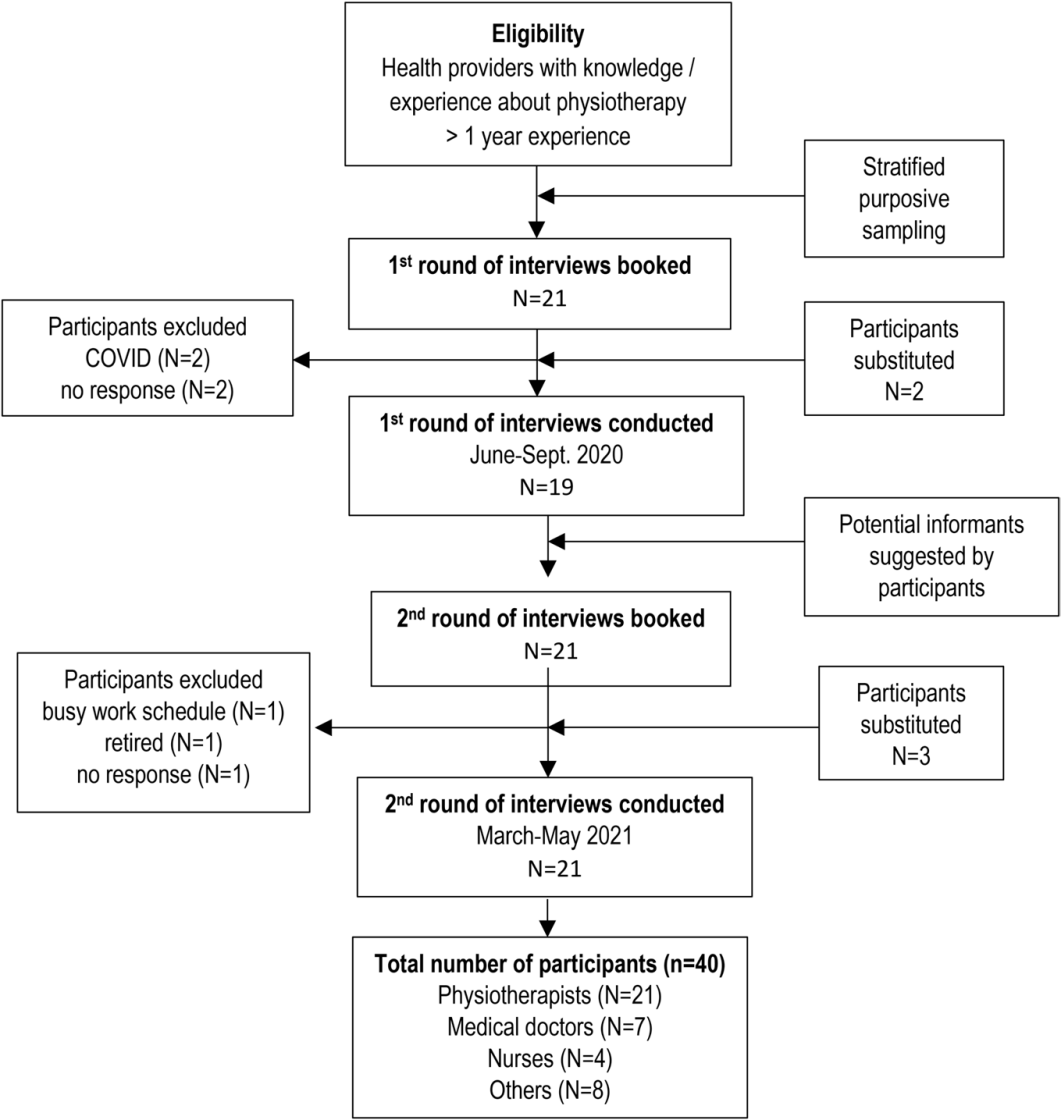
Recruitment procedure of informants

Participants were selected from Bagmati Pradesh, Province III of Nepal, the second most populated area including the capital Kathmandu to provide adequate representation of a variety of health professionals with different work settings and different backgrounds, including government representatives having a national overview of the situation. Inclusion criteria were a minimum of one year of working experience and having knowledge about and experience with physiotherapy services in Nepal [43]. Seven of those contacted declined due to: a busy work schedule, COVID, retirement, or no response. Missing participants were substituted (Fig. 1). There were no further barriers in the recruitment process.

### Ethical considerations and data security

Approvals were granted by the Nepal Health Research Council (NHRC-ERB Protocol No. 455/19), the Institutional Review Committee of Kathmandu University School of Medical Sciences / Dhulikhel Hospital (IRC-KUSMS 104/18), and the Norwegian Centre for Research Data (NSD 383,963). Informed consent was received from each participant before the interview with the right to withdraw from the study at any time. Situational and relational ethical challenges were considered. The researcher, being a physiotherapist and resident of the Bagmati Province III, was aware of the potential influence on the interviewees as either colleagues in academia, leaders, or clinicians. For the former, familiarity may have influenced the interview. For the latter, in a hierarchical system such as in Nepal, the inequality in social status between the participant and the researcher would sometimes put the researcher above and sometimes below the interviewee, depending on the position. The participants were assured they were not obligated to answer questions with which they felt uncomfortable. Personal information was kept confidential and audio recordings were securely stored in a folder only accessible to the researcher. No personal identifiers were mentioned in the digital files. Data was securely stored on the university server. Handling of personal data was following the EU General Data Protection Regulation (GDPR). The study was performed in accordance with the Helsinki Declaration.

### Data acquisition

Due to pandemic restrictions, all interviews were performed online using the university licensed Zoom compliant with GDPR (https://explore.zoom.us/en/gdpr/). Online interviews have been shown to be viable and satisfactory in qualitative research [44]. Written instructions and technical information were provided in advance to improve participant’s attendance. Interviews were conducted from multiple sites and different health professions to increase informational power [45, 46]. Interviews were conducted by the PhD candidate (NRS) in Nepali and undertaken from June 2020 to May 2021.

A generic guide for semi-structured in-depth interviews was developed (Supplement 1). Guiding questions targeted perceptions about disability and rehabilitation, and the role of physiotherapy in health promotion and prevention. Experiences of barriers and facilitators for physiotherapy services in healthcare in Nepal were probed to gain in-depth information. The guide was piloted in five interviews with different health professionals and necessary modifications were applied [47]. Pilot interviews were excluded from the data material.

### Data analysis

**Fig. 2:**
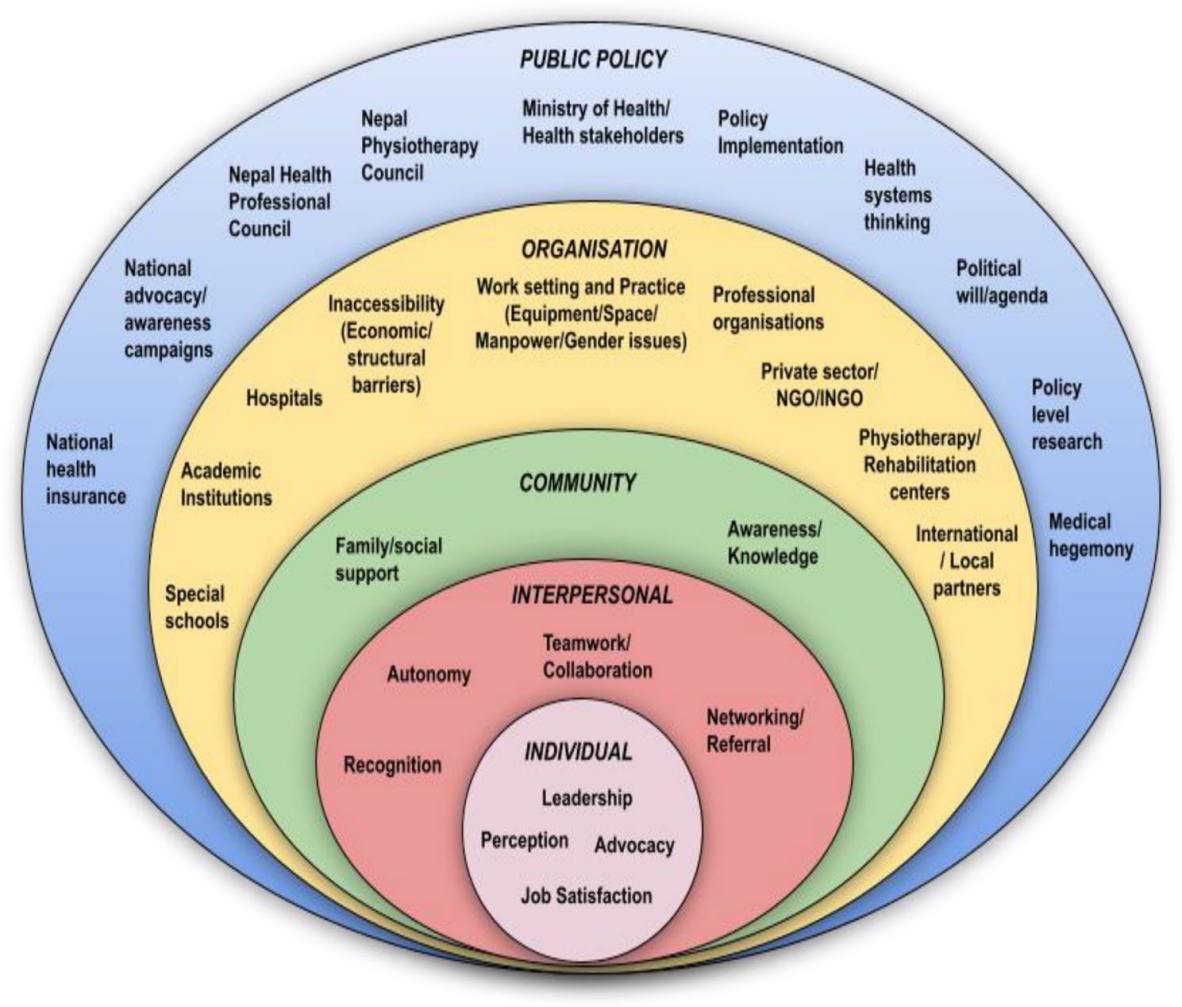
Socioecological framework for factors affecting physiotherapy services in Nepal. We used the socioecological concept [33] and constructed a five-level bubble diagram which we populated with categories under the themes derived from our interviews. The figure was redrawn using data acquired from different sources to guide the research. Factors included in the original model by McLeroy et al. Ecological perspective on health promotion programs, 1988. P355: *Individual* - Attitude, behaviour, knowledge, perception, self-concept, skills; *Interpersonal* - Formal social network and support system, Work group network, Friendship network; *Community* - Informal network within defined boundaries, Personal friendship network, Neighbourhoods; *Organisational* - Institutions with organisational characteristics and formal (and informal) rules and regulations for operation; *Public policy* - Local/State/National legislatures. *Note:* Barriers and facilitators for development of physiotherapy defined under different categories using thematic analyses were placed into the different levels in the socioecological framework based on contextual relation and significance to the Nepalese physiotherapy system or physiotherapy in general

All recorded interviews were transcribed verbatim in Nepali and translated to English. Accuracy of transcriptions, translations, and sufficient de-identification of content was secured. A codebook was prepared [48] and NVivo (QSR International, version 12, Release 1.5.2) was used for data management. Following the six steps of thematic analysis, we used a 15-point checklist of criteria to generate initial codes that clustered relevant texts into potential categories, themes and sub-themes which were reviewed, discussed and agreed upon [49]. NRS read all the transcripts and identified the possible codes from the pilot interviews initially and added a set of selected priori codes derived from previous studies and literature to develop a codebook. Further, NRS and AE (experienced in qualitative methods) independently and simultaneously coded the interview contents, and discussed and agreed as the new codes emerged. NVivo files were exchanged and merged in the continuing process. Frequent meetings and discussions were held with the team members (BMK and AKS). Categorising of the relevant texts generated a high number of loose, low-level codes and a large volume of potential quotes (around 800). These codes were clustered into potential themes and sub-themes by NS and AE. Other members on the research team (AKS and HM) reviewed the codes and themes to provide feedback and validation of the findings. Synthesis to higher level themes was achieved through extensive and critical review and discussion by the entire research team. These were placed into the five different SEM levels (Fig. 2). While thematic saturation was considered satisfied across SEM levels, the authors were aware that unique information from leaders may not be considered saturated as only one single or few persons would populate such positions. The concept of information power for evaluating sufficiency of the interview findings was applied [50]. We used an abductive reasoning approach to gain insights from our data [51].

## Results

Forty interviews were included in the analysis (mean duration 56 min, range 27–90). The majority were physiotherapists with a master’s degree having 2–11 years of work experience. For all participants, the mean age was 42 years and 14 (35%) were female (Table 1). The interviews identified barriers and facilitators, coded, and grouped into themes (Table 2), and categorized in accordance with the SEM levels [35, 38, 52] (Fig. 2). The analysis indicated that saturation might not be reached for some professionals as they had unique positions. Their contribution was still valuable for informational power and to address all SEM levels, particularly on public policy level.

**Table 1:** Demographic characteristics of participants

Demographics	*N* (%)
**Age**	
24–33	11 (27.5)
34–43	13 (32.5)
44–53	8 (20)
54–63	6 (15)
64–74	2 (5)
**Sex**	
Male	26 (65)
Female	14 (35)
**Education**	
Bachelor	12 (30)
Master	22 (55)
PhD	5 (12.5)
Post doc	1 (2.5)
**Profession**	
Physiotherapists	21 (52.5)
Doctors	7 (17.5)
Nurses	4 (10)
Social activist	3 (7.5)
Senior health administrators	2 (5)
Public health officer	1 (2.5)
Educationist	1 (2.5)
Registrar	1 (2.5)
**Participant’s overall years of experience**	
Years	
2–11	25 (62.5)
12–21	10 (25)
22–31	2 (5)
32–41	2 (5)
42–51	1 (2.5)
**Physiotherapist years of experience(** ***n*** ** = 21)**	
2–11	18 (85.7)
12–21	2 (9.5)
22–31	1 (4.8)

**Table 2:** Examples of barriers and facilitators with example quotes and themes. Barriers and facilitators belong to the corresponding category. The table and the following result section is built on the thematic analysis aligned to the levels in socioecological model

Individual level				
Example quotes	Barriers	Facilitators	Categories	Themes
“…Physiotherapy services are focussed on orthopaedic conditions at many hospitals…but there are different conditions and diverse scope in which physiotherapist should have been involved.” -*P2*, *Physiotherapist*	Lack of awareness of opportunity for expanded scope of practice	Positive self-concept about oneself (profession)	Knowledge, perception and self-concept	Physiotherapists as agents of change and leaders
“.we have submitted many documents about physiotherapy, but they were not addressed because we do not have physiotherapists at policy level. We must aspire to reach higher to uplift our profession [position].” -*P1*,* Physiotherapist*	Difficulties in advocacy	Commitment towards improving profession	Advocacy efforts	
	Lack of leadership positions	Physiotherapists’ taking initiation	Leadership attitude	
“There used to be more than 70 patients…hospital promised us overtime payment which we never received…should never happen to young physiotherapists like us. But we boldly kept this issue in the board meeting.” -*P8*,* Physiotherapist*	Individual negative experiences	Raising the voice/ Effort/ Skill to address the issues	Personal experiences	
**Interpersonal level**				
“You can go home and exercise yourself. You don’t have to go elsewhere.” Patients are given this sort of advice. So, there is a lack of knowledge in doctors at some centres.” - *P9*,* Physiotherapist*	Having low or negative attention/ Lack of autonomy	Understanding the significance of physiotherapy by other professionals	Formal networking/ Teamwork	Biomedical model and its influence on interaction between health professionals
“Unless physiotherapist establish networks and provide quality service, it is difficult for us to gain the trust of the stakeholders, patients and doctors.” -*P5*,* Physiotherapist*	Lack of recognition	Gaining positive attitudes and respect	Establishing professional network	

*NB*: The following descriptions were informed by the results under each level presented as in the example and too long to include in this paper

### Individual level

Factors perceived at the individual level affecting physiotherapy services necessitating leadership skills.

### Physiotherapists as agents of change and leaders

#### Misunderstanding and limited insight about physiotherapy in healthcare

Participants generally expressed a lack of knowledge of the physiotherapist’s professional role in prevention, health promotion, and well-being.

*“I think the role of physiotherapy in health promotion has not been clearly understood by both physiotherapists and patients. Patients think they need it only when they have paralysis or pain*,* back pain.……. it is important that we as a physiotherapist should first explore a little bit.” (P3*,* Physiotherapist)*

#### Taking the lead and advocacy both as challenges and opportunities

The predominant barriers experienced by several participants were a lack of influential positions and advocacy for change. A physiotherapist *(P1)* stated that they were not informed about meetings with key stakeholders for important planning and decision-making. This was corroborated by non-physiotherapists describing that the lack of key positions reduced physiotherapists’ opportunities to be a part of policy decision-making and advocacy for their important role in the healthcare system.

*“….if you look at the higher post in the hierarchy*,* where physiotherapists are now in the professional stand plays a great role. Where are physiotherapists in the government?” (P22*,* Senior public health officer)*

A policy maker from the Health Ministry and a physiotherapist (*P29*,* P2*) mentioned in contrast that physiotherapy representation was either irregular or lacking in important high-level meetings, despite invitations. They explained that physiotherapists should develop better communication skills and understand the context and health system of Nepal before putting forth professional agendas in high-level meetings. Others mentioned that physiotherapists are lagging in taking the lead in advocating for their profession and its expansion possibilities. The reasons, one senior physiotherapist stated (*P2*), were fear of increasing their workload, fear of approaching doctors or lacking advocacy skills. Consistent advocacy, communication, and leadership skills were identified as keys to change and improvement. Several participants remarked that advocacy was promoted through individual efforts and networking outside of the established healthcare system. While the establishment of private physiotherapy clinics and rehabilitation centres are increasing in the cities, one physiotherapist *(P3)* described that success depends on individual vision and motivation, leadership skills, and multidisciplinary teamwork. Another participant noted that the development of physiotherapy outside the established system is a consequence of lack of responsibility taken by the concerned authorities or government.

#### Individual experiences influence actions impacting the services

Several physiotherapists described that individual experience with the hospital systems and work culture affects the level of job satisfaction. A newly graduated physiotherapist (*P8*) stated that they would strive for change in the workplace according to what they have learned and practiced from education. The work culture represented by senior physiotherapists or department leaders was however described as challenging to their expectations. Several physiotherapists related low job satisfaction to low wages, limited professional opportunities and confusion about careers and the future. These factors were proposed as contributing to “brain drain”. Low remuneration was described as insufficient for making a living on the salary the physiotherapists receive.

*“If you cannot earn a motorcycle or run a life or family in a city*,* it will be very difficult to sustain for people with this profession and … get diverted slowly. So why to study physiotherapy?” (P1*,* Physiotherapist)*

##### Interpersonal level

Effects of networking with different health professionals for positive impact on the physiotherapy services and patient’s beliefs.

### Biomedical model and influence from other health professionals

#### Establishing networks for advocating physiotherapy services

Some participants explained that collaboration was about mutual motivation and action between physiotherapists and other medical professionals. It was suggested that physiotherapists should be proactive. Many physiotherapists experienced their role as being unrecognized by other health professionals, the government and the public. While there are improvements, a few physiotherapists pointed out that there are still doctors who would prescribe exercises to patients rather than referring to a physiotherapist. Some stated that physiotherapy still resided under the department of orthopaedics despite its wider scopes of practice, thus receiving mainly referrals for musculoskeletal conditions. A few described that many patients are not concerned about the importance of a healthy lifestyle, instead they request quick fix options e.g., pursuing medication after the physiotherapy consultation due to health belief models promoting use of medication. Several participants described involving healthcare stakeholders for teamwork and collaboration as facilitators for acknowledging physiotherapists in different settings.

*“…. we thought that physiotherapy is done only after discharge but when I worked at (…) hospital as a disaster focal person during earthquake*,* then I understood about the importance of physiotherapy.” (P24*,* Nurse)*

##### Community level

The community impact on physiotherapy and health services and the need of communication.

### Building trust, awareness, and support from society for service utilisation

#### Patient/public awareness about physiotherapy

Most of the participants expressed that awareness and knowledge about the healthcare system, physiotherapy and rehabilitation would significantly impact health-seeking behaviour. Lack of knowledge was described as contributing to unequal access to services, receiving no or wrong treatment and reinforcing medical dominance.

“*We have a patient here*,* a very young patient who is just 23–24 years old. He had SCI [spinal cord injury] five years ago and he did not know what to do*,* where to go. So*,* he stayed at home for a long time*,* he was an incomplete [SCI] patient. Had he come to us at the right time*,* he would have been a lot better.” (P9*,* Physiotherapist)*

#### Awareness of appropriate service delivery for health needs

Some participants experienced the community’s failure to give health service support to people with disability and their families. This ultimately led to uncertainty for patients, embarrassment from society, and fear of disclosure about the disabled family member, potentially resulting in a life in isolation for them.

“*I deal with paraplegic cases in players [sports]. First*,* it is difficult [for patients] to reach the clinic*,* second is the support from family and respect that they should have got from society. It may be difficult for patients to come by themselves or not willing to come or that feeling what society would think about them.” (P1*,* Physiotherapist)*

#### Cultural beliefs, family, and social systems influencing access and service utilisation

Participants described how they perceived different perspectives on disability and health promotion in the general population. Negative perceptions or insufficient understanding were considered a barrier to advancing physiotherapy services. A physiotherapist (*P2*) described that society and involved authorities only considered disability when long-term and severe. Another physiotherapist and a doctor *(P25*,*34)* recognized how the views on people with disability impacted their life choices and capabilities. Some were also concerned that the government’s perception of disability led to inadequacy in rehabilitation policies. Stigmatization and discrimination of people with disability by both family and society were reported as a challenge resulting in barriers to accessing required treatment and support. The participants shared their experience with people in the community about the belief that *“disability is the result of the sin of past life…” (P38*,* Social activist)*,* “as a blame shifting and torture to female*” *(P12*,* Physiotherapist).* Attitude towards gender discrimination was highlighted as a challenge for equal accessibility to service. Information was considered important for raising awareness and developing trust concerning the impact of treatment for people with disabilities.

*“They [people] have thoughts like it is not worth providing services as she is just a daughter*,* with deformed arms and legs after an accident…. a difficult situation for women and girls to go there [Rehabilitation centres] with family support and financial constraints” (P38*,* Social activist)*

##### Organisational level

Challenges regarding affordability and accessibility including geography, remoteness, infrastructure, organisational roles and opportunities.

### Challenges and disparities in various settings

#### Financial, geographical, and structural challenges to physiotherapy services

Structural and economic challenges were highlighted as significant barriers to health services. Most participants reported challenges for affordable services in rural settings where people seek either “freebies” or inexpensive services. Many explained that “*patients feel a lot of financial burden*” and that expensive treatments lead to unsatisfactory care and rehabilitation outcomes for patients unable to afford treatment. The lack of government supported rehabilitation centres in combination with expensive services in the private sector was described as putting pressure on the families’ economy.

*“It [Rehabilitation] takes a long time*,* and family has a big financial burden. The family must pay a high price because there is no rehabilitation centre built by the government and the rehab centres opened in a private set up are very expensive.” (P27*,* Doctor)*

The participants described differences in accessibility to physiotherapy services in rural vs. urban areas. The lack of physiotherapy facilities in rural areas was described as due to “*centralisation*” and geographical barriers. Unreachable areas due to difficult terrain and difficulty in transportation have negative consequences for patients.

“*Health service accessibility is very low in the Himalayan and high mountainous region*,* compared to flat terai regions and newly developed urban areas.” (P40*,* Doctor)*

Some participants shared their experience of lacking support from the family and a lack of disabled-friendly infrastructure to visit the health facilities. They also described that “*walking for hours*” was a problem in reaching the facilities which had a negative impact on treatment and follow-up. Rural areas are left behind due to a lack of physiotherapy facilities and people in these areas are therefore not considering physiotherapy. In contrast, people in urban areas were described to, in general, having sufficient access to rehabilitation services which highlights the unequal access to rehabilitation services in Nepal.

*“If there are some people with disabilities in this remote area*,* it is very difficult for them to come to this centre and it is also difficult for us to reach there.” (P11*,* Physiotherapist)*

#### Challenges at work settings and the clinical practice

Participants, mainly physiotherapists, frequently reported work-related issues in clinical practice. Lack of resources such as equipment, adequate workspace and manpower were described to be some of the main barriers. Other barriers were deficient evidence-based practice, insufficient expertise, and lack of physiotherapists with specialisations. Specialisation such as women’s health, paediatrics, sports rehabilitation, cardiorespiratory physiotherapy, mental health and geriatrics, were mentioned. High patient turnover was reported as an issue in in-patient departments resulting in early discharge cutting short the necessary hospital-based rehabilitation. Lack of time was reported to limit teaching the patient necessities such as eating and dressing to independently manage daily living activities at home.

*“The number of patients used to cross over 70. We had to finish our lunch quickly and attend the patients as soon as possible. I worked this way probably for five months. The hospital promised us overtime payment as recorded in our logbook*,* but we never get paid.” (P8*,* Physiotherapist)*

#### Challenges for establishment and regulation of academic institutions

Many participants acknowledged the physiotherapy programs of Kathmandu University and Pokhara University and additional upcoming programs at other institutions. However, only two (*P37*,* P39-both doctors*) mentioned the challenges for the establishment and regulation of such educational institutions due to rigid academic rules, regulations and credentials. There are a limited number of seats available in the few physiotherapy colleges in Nepal. Participants reported that those who manage to get admitted and aspire to pursue a physiotherapy career often are left with no adequate job opportunities after graduation. Physiotherapists graduating with a bachelor’s degree have no option for postgraduate studies in Nepal. In both cases, young Nepalese physiotherapists are compelled to move abroad.

*“I have done only a bachelor’s degree*,* and it is around 10 years now*,* but due to my personal reasons*,* I have not been able to go abroad to pursue my master’s degree. If it had been available in our own country*,* many friends and I would have done it in Nepal.” (P1*,* Physiotherapist)*

#### Efforts from professional organisations and the private sector

Study participants commended efforts made by professional organisations like Nepal Physiotherapy Association (NEPTA) for advocacy, leadership, awareness programs and scientific and continuous professional development programs, leading to a shift in professional recognition and development in Nepal. Non-government organisations were also identified as facilitating contributors in the disability and rehabilitation sectors. However, one physiotherapist and a social activist (*P6*,* P38*) raised a serious concern about the increasing financial burden to such organisations for sustainability and that the government should provide financial assistance. A few participants also acknowledged the government’s support of disability care in public hospitals and special schools. Concerns were however raised about lack of adequate financial support, absence of monitoring and evaluation of improvement for people with disability and non-existent standard protocols for running the special schools in Nepal. There were also concerns about the absence of regulations for establishing physiotherapy clinics, resulting in practices with insufficient equipment and displaying conduct to earn quick investment returns for the business rather than quality services. One senior physiotherapist (*P2*) considered this as a threat to the way the profession and services are expanding.

##### Public policy level

Health policy, decision-making, implementation, good governance, health systems approach, and research to increase the support and coordination of services.

### Health politics and structures

#### Prioritisation and political will to increase the support from the government and stakeholders

Many participants mentioned a lack of awareness of physiotherapy at the government level and that physiotherapy is not considered important and is often underestimated. A physiotherapist and a doctor *(P2*,* P40)* described the importance of incorporating physiotherapy into the political agenda of political parties during their election campaigns as they do for other health issues. Several participants viewed the government as disinterested, without political will and a lack of commitment to advancing physiotherapy services.

*“In many hospitals*,* provincial governments have opened vacancies for other health professionals at every root level*,* but physiotherapy is not available.” (P3*,* Physiotherapist)*

In contrast, some of the participants acknowledged increased support and recognition from the government after the earthquake in 2015 and emphasized the need for additional advocacy. Many agreed on what a supportive government response should endorse: “*speaking up about physiotherapists’ significance in the healthcare system*,* creating public vacancies*,* and making leaders within governing bodies and ministries aware of the role of physiotherapy*”.

Including physiotherapy services as part of the health insurance coverage was perceived as a facilitator *(P30*,* Senior health administrator)*, generating increased referrals for healthcare. A senior health administrator *(P31)* disclosed that the government has initiated a Health Management Information System (HMIS) for collecting and compiling data for monitoring, evaluation, and management of health service delivery information. HMIS covers all health facilities with physiotherapy and other rehabilitation services and NEPTA is expected to support and spread information about HMIS.

#### Need for strong governance, health system’s thinking, and research

Several of the physiotherapists had concerns that NHPC is not effectively monitoring and regulating clinical practices and services. They mentioned that a separate Nepal Physiotherapy Council is an urgent need to address the alarming situation of unauthorised and unethical practices and mis-regulation to establish physiotherapy as a dignified and valued profession. It was also mentioned that the government prioritised infrastructure and buildings and perceived physiotherapists’ role as mainly curative, resulting in a lack of health promotion investments.

*“. the government still has not fully understood about prevention. The Ministry considers that the hospitals are needed to make citizen healthy. Hospital is a kind of facility where people come only when they get sick.” (P34*,* Doctor)*

Emphasis was placed on the need for “health system’s thinking”. A lack of holistic, coordinated, and realistic approach toward healthcare was perceived as a barrier among the policymakers hindering policy implementations. Several participants *(P22-Senior public health officer*,* P32- Senior nurse*,* P34*,* P40- Doctors)* described medical dominance and superiority as a challenge to other health professionals’ development.

*“There must be a coordinated action amongst councils*,* associations*,* academics etc. There is a medical dominance in the health sector. We need to discuss how to have integrated team in all sectors.” (P22*,* Senior public health officer)*

*A senior health administrator* (*P30*) suggested exploring and conducting economic evaluations of rehabilitation services to inform policymakers. Some participants considered the need for research utilisation, specific treatment guidelines and evidence-based practice to provide quality treatment. However, transferring the knowledge into practice or a policy brief was considered a challenge.

*“Our colleagues are publishing in good journals. We are not translating them into practice or in the form of a policy brief*,* so there is an information barrier.” (P2*,* Physiotherapist)*

## Discussion

This study used a socioecological framework (SEM) to recognise multilevel factors affecting the development of physiotherapy services in Nepal. Identifying barriers and facilitators at multiple levels enables actions targeting system-wide changes. Our findings revealed factors on all five SEM levels. Most were framed as barriers whereas some may be amendable and turned around as facilitators. Our stratified, purposive sampling approach generally ensured maximum variation of perspectives, although saturation for some information obtained from leaders and policymakers might be compromised due to a limited number holding such positions. Some factors may have fitted in at all or different SEM levels. For instance, leadership and advocacy efforts were recognised mainly as skills and experiences at the individual level but could also be considered as interpersonal and shared experiences, or something necessary at the community or organisational level.

At the *individual level*, lack of understanding and awareness of scope of physiotherapy services were significant factors affecting the services. Low job satisfaction among physiotherapists was mentioned as a significant risk factor for “brain drain” corroborating previous studies in Nepal [9, 53] resulting in a lack of human resources in the health system [11, 27, 54]. The importance of advocacy and communication skills to raise the profession’s profile and status in the context of evolving healthcare systems highlighted by the study participants is well supported [3, 55, 56]. Lack of communication and awareness among health professionals and communities are obvious barriers to development [57]. As only 17% of medical interns in a Nepal hospital have adequate knowledge about physiotherapy [58], this demonstrated the needs for improved communication and awareness of physiotherapy. Individual efforts of advocacy and leadership may contribute to increased awareness although should ideally be addressed at the organizational or policy levels for wider effect.

At *the interpersonal level*, our findings suggested the need for networking and maintaining close working relationships with medical professionals [3], especially doctors, for positive influences on physiotherapy services. While the Nepalese culture is gradually adopting a biomedical healthcare approach [59, 60], interdisciplinary approaches could be strong facilitators promoting a biopsychosocial view. Our participants acknowledged gradual changes in other health professionals’ attitudes towards the profession. At the *community level*, we found that various beliefs and social systems negatively impacted service access and utilization. To improve this, our study, in line with others, highlighted the need for campaigns and community-based programs to advance awareness and health promotion [11, 61]. Focus on the community level [14, 61] is crucial as supported by a recent survey revealing that ~ 46% of the children with cerebral palsy in Nepal have never received rehabilitation, mainly due to lack of awareness [62].

Factors at the *organisational level* were described for the unsatisfactory situation where unequal and difficult access to health- and physiotherapy services stood central, corroborating similar findings from studies in Nepal [19] and other LMICs [30, 31, 63]. Our study pointed out that many patients cannot reach or afford treatment. Geographical infrastructure will remain a challenge [64], and with financing of UHC posing a major issue [16] expansion of physiotherapy remains inhibited, especially in rural areas. As a result, people in low resource settings encounter limited or absent access to health services and rehabilitation [11]. In line with others, our study indicates that a lack of general assets and human resources negatively affects both the work environment and the quality of services [61, 65, 66]. Government initiatives and efforts from activists, and organisations, particularly NEPTA, were seen as facilitators. Considering the rapid shift in health systems structure in Nepal [67], the participants emphasized that professional organizations must work at a quicker pace and remain alert about the system changes. The importance of the educational sector in these areas is crucial and cannot be disregarded. Our interviews revealed that lack of career opportunities and higher academic advancements are causing many ambitious physiotherapists to leave Nepal for opportunities abroad. This finding is corroborating the World Physiotherapy interview report [68]. At the same time, the physiotherapy workforce in Nepal is well educated, but at an initial career state [9]. Altogether there are six colleges offering bachelor programs adding up to a total of 150 seats [69]. In the Fall 2022, the first master in physiotherapy was accredited [70].

At *public policy level*, flaws in implementation, lack of commitment and interest in physiotherapy as a public health priority were major concerns. In line with others, our study supported that advocacy efforts at all levels were considered facilitators [11, 13, 71]. The Nepal Policy Strategy and 10 Years Action Plan on Disability Management 2016–2025 [72] aims to prevent disability among citizens and ensure equal access to promotive, preventive, curative, and rehabilitative health services for people with disabilities. However, constant changes in leadership, ministers and authorities make it complex for follow-up of advocacy and development agendas [73]. Without clear government policy documents from the Ministry of Health and Population declaring physiotherapy as an obligatory service in the health system, action is not taken. A positive aspect is the World Health Organization consensus report stating that rehabilitation services should be integrated at all levels of the health system [54]. In agreement with a similar study [63], several participants said that this requires proper policy planning and implementation with a holistic approach and health systems perspective. Our study strongly supported physiotherapists’ role in health promotion activities in Nepal, as emphasized in the Global Health Summit papers [3, 74]. It also underlined the importance of evidence-based practice, clinical practice guidelines and implementation as necessities to provide quality and patient-centred care. Several previous studies have however highlighted challenges impacting physiotherapists’ ability to follow and implement clinical practice guidelines due to lack of remuneration, support, manpower, high workload, and lack of research- and analysis skills [75, 76].

### Strengths and limitations

A strength of this study was the extensive interviews of health providers across professions and hierarchies and the use of SEM as a framework. SEM proved useful for categorising identified facilitators and barriers and providing a comprehensible overview. Saturation was however not considered reached at all levels due to that top leaders are limited in numbers. A limitation was that interviews did not include the views directly from the patients or the public but were information conveyed by the study participants. This study reflects the situation when data was collected. Although the situation in Nepal is changing, the authors believe that the general results are valid.

## Conclusion

Overall, our study highlights the need for addressing barriers and leveraging facilitators at multiple levels to strengthen physiotherapy services in Nepal. Efforts from individuals, organizations, policymakers and the government are crucial for improving access, awareness and quality of physiotherapy services in the country. Considering the prevailing situations and implementation status as of now, we consider the results of our study as valid and applicable to Nepal. Our broad and structured investigation strategy is applicable to others for a comprehensive analysis of barriers and facilitators for providing information for the development of physiotherapy by pointing out key elements needing attention.

### Electronic supplementary material

Below is the link to the electronic supplementary material.

Supplementary Material 1

Supplementary Material 2

Supplementary Material 3

## Data Availability

Data supporting the findings of this study are available in the manuscript or supplementary materials. Interview data is not available for sharing.

## Declarations

### Authors’ information

The authors involved have varying levels of professional qualifications. NRS is a senior physiotherapy academician and clinician at KUSMS, former president of NEPTA and a Ph.D. scholar at NTNU. GW has worked with development of physiotherapy in South-East Asia for more than 20 years. AE was a physiotherapy master graduate with experience in qualitative studies and currently a Ph.D. scholar at NTNU. HM and BMK have strong qualitative research background. All the authors have significant engagement in global health especially in health promotion, non-communicable diseases, disability, physiotherapy and rehabilitation. All the authors have a physiotherapy background, while BMK is the director of the Public Health, Community Programs and Global Engagement at KUSMS and administrative director of Dhulikhel Hospital, Nepal.

## Abbreviations

GDPR: General Data Protection Regulations
HMIS: Health Management Information System
KUSMS: Kathmandu University School of Medical Sciences
LMIC: Low- and Middle-Income Countries
NEPTA: Nepal Physiotherapy Association
NHPC: Nepal Health Professional Council
NHRC: Nepal Health Research Council
NTNU: Norwegian University of Science and Technology
UHC: Universal Health Coverage
