# Supplementary material for: Barriers and facilitators for strengthening physiotherapy services in Nepal: perspectives from physiotherapists and health providers
Source: BMC Health Serv Res. 2024 Aug 1;24:876. doi: 10.1186/s12913-024-11272-w (PMC11295310; doi:10.1186/s12913-024-11272-w)
Supplement: Supplementary file 1 — Supplementary Material 1 [file 12913_2024_11272_MOESM1_ESM.docx]

|  | How would you define *disability*? |
| --- | --- |
|  | How would you define *rehabilitation*? |
|  | How do you view the current level of physiotherapy services and disability care in your institution/organization? |
|  | What do you think is the current level of physiotherapy services and disability care nationally?  Probes: In relation to scope of services, quality, cost, reach and access  Has the (disability) physiotherapy service reached to those in need? |
|  | What do you consider are the major barriers/challenges to develop physiotherapy services and disability care in Nepal?  Probes: society, organization, community, interpersonal relationships and individual level; Awareness, Policy, Financing, Cultural issues |
|  | What do you consider are the major facilitators/opportunities to develop physiotherapy services and disability care in Nepal? |
|  | What is your opinion on prevention and health promotion?  Probes: Is/should it be part of physiotherapy? |
|  | What do think the role of physiotherapy is for prevention and health promotion? |
|  | Should the role of physiotherapy be different, if so - how (considering the needs)? |
|  | Do you think there is a need for post graduate education in physiotherapy, if so, what should it prioritize? |
|  | Should physiotherapists hold higher positions in health politics for advocacy? |

Interview questions
