# Supplementary material for: Barriers and facilitators for strengthening physiotherapy services in Nepal: perspectives from physiotherapists and health providers
Source: BMC Health Serv Res. 2024 Aug 1;24:876. doi: 10.1186/s12913-024-11272-w (PMC11295310; doi:10.1186/s12913-024-11272-w)
Supplement: Supplementary file 2 — Supplementary Material 2 [file 12913_2024_11272_MOESM2_ESM.docx]

**Table a:** Participants code with different professional backgrounds

| **Participant code** | **Profession** |
| --- | --- |
| P1-P19, P25, P35 | Physiotherapist |
| P20,P26,P27,P34, P37,P39,P40 | Doctor |
| P21, P24, P29, P32, | Nurse |
| P23, P33, P38 | Social activist, Disability organisation representative |
| P30 | Senior health administrator |
| P31 | Senior health administrator, Ph.D. |
| P22 | Public health officer, Ph.D. |
| P28 | Educationist, Ph.D. |
| P36 | Registrar |

Note: The professionals are a mixture of clinicians, academicians, member representatives of medical commissions, councils, professional associations, ministry, disability associations, public and private sector, and international agencies

| **Table b**: Participants’ main affiliation and different facilities | | |
| --- | --- | --- |
| **Facilities** | **Number (%)** | **Participant codes** |
| Council/ Commission/  Association | 10 (25%) | P1, P5, P10, P21, P22, P32, P33, P36, P38, P39 |
| Academics | 6 (15%) | P16, P20, P23, P34, P35, P37 |
| Government | 10 (25%) | P4, P11, P19, P24, P25, P27, P29, P30, P31, P40 |
| Private | 6 (15%) | P3, P8, P13, P15, P17, P18 |
| Specialised Rehabilitation Centres/ /NGO/INGOs | 8 (20%) | P2, P6, P7, P9 , P12, P14, P26, P28 |
| Total | 40 (100%) |  |

Councils and commissions: MEC, NHPC, NMC, NNC Associations: NAN, NEPTA, NMA, NFDN

MEC: Medical Education Commission NEPTA: Nepal Physiotherapy Association

NHPC: Nepal Health Professional Council NAN: Nursing Association of Nepal

NMC: Nepal Medical Council NMA: Nepal Medical Association

NNC: Nepal Nursing council NFDN: National Federation of the Disabled-Nepal

NGO: Non-Government Organisation

INGO: International Non-Government Organisation
