# Supplementary material for: Barriers and facilitators for strengthening physiotherapy services in Nepal: perspectives from physiotherapists and health providers
Source: BMC Health Serv Res. 2024 Aug 1;24:876. doi: 10.1186/s12913-024-11272-w (PMC11295310; doi:10.1186/s12913-024-11272-w)
Supplement: Supplementary file 3 — Supplementary Material 3 [file 12913_2024_11272_MOESM3_ESM.docx]

| **Table 2.a: Examples of barriers and facilitators with example quote and themes** | | | | |
| --- | --- | --- | --- | --- |
| ***Example quotes*** | ***Barriers*** | ***Facilitators*** | ***Categories*** | ***Themes*** |
| ***Individual level*** | | | | |
| *“...physiotherapy services are focussed on orthopaedic conditions at many hospitals…but there are different conditions and diverse scope  in which physiotherapist should have been involved.”-P2* | *Lack of awareness of opportunity for expanded scope of practice* | *Positive self-concept about oneself (profession)* | *Knowledge, perception and self-concept* | *Physiotherapists as agents of change and leaders* |
| *“..we have submitted many documents about physiotherapy, but they were not addressed because we do not have physiotherapists at policy level. We must aspire to reach higher to uplift our profession [position].” -P1* | *Difficulties in advocacy* | *Commitment towards improving profession* | *Advocacy efforts* |  |
|  | *Lack of leadership positions* | *PTs taking initiation* | *Leadership attitude* |  |
| *“There used to be more than 70 patients…hospital promised us overtime payment which we never received…should never happen to young PTs like us. But we boldly kept this issue in the board meeting.” -P8* | *Individual negative experiences* | *Raising the voice/ Effort/ Skill to address the issues* | *Personal experiences* |  |
| ***Interpersonal level*** | | | | |
| *“You can go home and exercise yourself. You don’t have to go elsewhere.” Patients are given this sort of advice. So, there is a lack of knowledge in doctors at some centres.”- P9* | *Having  low or negative attention/ Lack of autonomy* | *Understanding the significance of physiotherapy by other professionals* | *Formal networking/ Teamwork* | *Biomedical model and influence from other health professionals* |
| *“Unless physiotherapist establish networks and provide quality service, it is difficult for us to gain the trust of the stakeholders, patients and doctors.” -P5* | *Lack of recognition* | *Gaining positive attitudes and respect* | *Establishing professional network* |  |

| **Table 2b**. **Examples of barriers and facilitators with example quote and themes** | | | | |
| --- | --- | --- | --- | --- |
| ***Example Quotes*** | ***Barriers*** | ***Facilitators*** | ***Categories*** | ***Themes*** |
| **Community factors** | | | | |
| *“They raise their voice when paracetamol is out of stock, if their children do not get vaccines, but there is no voice when physiotherapy is not available in the hospital.” - P30*  *“Patients with diabetes and hypertension (in hospital) do not come to us for physiotherapy...”-P14* | *Lack of knowledge about health services and health system*  *Awareness of service delivery* | *Spreading awareness for the need of services* | *Patient/Public Awareness* | *Building trust, awareness and support from society for service utilisation* |
| *“I have my own family experience of bedridden patient for 5-6 months in. I've seen how the communication done by physiotherapy boosts the morale of an injured patient.: -P21* | *Influence of family and social systems on accessing services* | *Need for communication* | *Cultural beliefs, family, and social systems* |  |
| *“Disabled people are only viewed with sympathy. Even disabled people have not understood that they can live a productive life as well.” - P34*  *“Disability is always connected only with people having birth defects or ... that is due to the limited knowledge in Nepal.” – P25* | *Negative perception about disability*  *Gender barrier* | *Developing family and social support* |  |  |

| ***Table 2c. Examples of barriers and facilitators with example quote and themes*** | | | | |
| --- | --- | --- | --- | --- |
| ***Example Quotes*** | ***Barriers*** | ***Facilitators*** | ***Categories*** | ***Themes*** |
| ***Organisational level*** | | | | |
| ***Example Quotes*** | **Barriers** | ***Facilitators*** | **Categories** | **Themes** |
| *“All good services are in Kathmandu, there are hardly reliable hospitals in the periphery or remote regions of Nepal.” -P5* | *Financial and geographical challenges* | *Promoting facilities at underserved regions* | *Access to services* | *Challenges and disparities in various settings* |
|  | *Lack of services availability* | *Expansion of services* |  |  |
| *“Different important branches (specialties) of physiotherapy have not been provided with needed space, equipment, and manpower in Nepal..” -P36* | *Lack of workforce, equipment, space, manpower*  *Lack of quality services, remuneration, career options* | *Improving the resources*  *Need for monitoring and evaluation* | *Challengers at work settings and the clinical practice* |  |
| *“...most of the students are studying abroad as there are only 30-40 seats available. Those who do not get, there is no alternative except going abroad…”.-P16*  *“I think in another 10 years’ time, there would be few more physiotherapy colleges ..”-P35* | *Lack of educational opportunities* | *Strengthening educational programs and capacity* | *Educational opportunities* |  |
|  | *Lack of specialisation and experts* | *Collaboration and communication* |  |  |
| *"We need to have a shared leadership approach to drive or advocate the issues through associations like people with disability association...…." -P2*  *“The special schools (for disability) running in Nepal are lacking holistic approach… There is no proper monitoring and regulation of people with disability?” -P12* | *Profit oriented organisations or physiotherapy centres* | *Role of disability organisations and associations, special schools, Role of NEPTA*  *Role of NGO/INGO* | *Role of professional organizations and private sector* |  |

| **Table 2d**. **Examples of barriers and facilitators with example quotes** | | | | |
| --- | --- | --- | --- | --- |
| ***Example Quotes*** | ***Barriers*** | ***Facilitators*** | ***Categories*** | ***Themes*** |
| **Public policy level** | | | | |
| *“There are policies…., but when it comes to implementation, it is very poor”-P34* | *Lack of implementation* |  | *Prioritised policies and programs settings* | *Health politics and structures* |
| *“Having physiotherapist at the Ministry of health, provincial health directorate, disability section of Epidemiology and Disease Control division (EDCD) and at least at the secondary hospitals are something doable within 5 years.” -P2* |  | *Planning and executing the achievable goals* |  |  |
| *“Provincial governments have opened vacancies for other health professionals at every root level in many hospitals, but physiotherapy is lagging behind.” -P37* | *Lack of prioritisation* |  |  |  |
| *"Physiotherapy institutions should also start registering data in Health management information system. Data is very important for us. We are also working on the health insurance scheme for this sector." -P30* |  | *Integrating into national programs* |  |  |
| *“First and foremost, there is no realisation and dedication in a leader or at political level that it (physiotherapy) is an important part of the health sector.”-P30* | *Lack of commitment and support* | *Increase awareness at governing level* | *Political will* |  |
| *“Political parties make a manifesto of their election with different health propositions, but not for physiotherapy and rehabilitation.”-P40* |  | *Need for a political support* |  |  |
| *“If not at the central level, we can do it at the district level in government sectors. And it is also possible if the influential donor agencies like WHO, USAID…are convinced to start projects in rural municipality. ” - P40* |  | *Support from national, international agencies and government* | *Advocacy and support at the level of governing bodies and stakeholders* |  |
| *“Since the government itself has not sensitively taken the issue of disability, it is obvious that physiotherapy services are also not included as important ones.” -P33* | *Less priority for preventive and promotive strategy* | *Need for prioritisation, advocacy* |  |  |
| *“We saw that orthodontist as a general secretary in Health ministry, created many positions for dental sector. So, if the physiotherapists get higher positions, they would also have addressed their sector.” -P28* |  | *Higher positions in governing bodies for decision making* |  |  |
| *“There is no separate division or section to drive physiotherapy sector as for other health services”-P2* | *Lack of monitoring and regulation* | *Need for a proper regulating body* | *Need of strong governance* |  |
| *“…there is a lack of system’s thinking and rehabilitation diplomacy in rehabilitation service providers and health system itself...the sector is not able to plan and prepare policy that is acceptable, feasible and reliable.”-P2* | *Lack of proper planning and integration* |  | *Health systems thinking* |  |
| *“We are not able to establish rehabilitation as an important part of the health system as the developed countries have.”- P27* |  | *Need for a holistic approach* |  |  |
| *“There is significant medical dominance leading it to Ministry of disease rather than Ministry of Health.” -P34*  *“Doctors always think that other professionals are nothing. They think they are the superior one…” -P32* | *Power hierarchy, power dynamics, medical dominance* | *Need for coordinated action* | *Medical hegemony* |  |
| *“ We need research evidence in physiotherapy and rehabilitation. I do not know if there are such research on health economics in this sector to refer...” - P30* |  | *Promoting health policy research* | *Policy level research* | *Health systems research* |
| *“Physiotherapy research is mostly clinical, and lacks systems or operational research if we need to advocate. So, we use WHO and other references. We are not translating our research into practice or in the form of a policy brief, so there is an information barrier.” -P2* |  | *Implementation research/Translation research* |  |  |
